# Locally‐led maladaptation as a configuration of responsibilities: ethnographic photo essay of a bamboo wall in Bangladesh

**DOI:** 10.1111/disa.70044

**Published:** 2026-02-05

**Authors:** Hyeonggeun Ji, Rawnak Jahan Khan Ranon

**Affiliations:** ^1^ International Institute of Social Studies, Erasmus University Rotterdam The Netherlands; ^2^ International Centre for Climate Change and Development Bangladesh

**Keywords:** bamboo wall, Bangladesh, disaster risk creation, displacement, locally‐led adaptation, maladaptation, responsibility

## Abstract

The construction of *bandals* (bamboo walls) is a widely practised climate adaptation initiative in Bangladesh, embodying community agency. This article interrogates how it can also represent *locally‐led maladaptation*—adaptive efforts that inadvertently sustain or exacerbate the very risks they seek to address. Drawing on ethnographic fieldwork in a riparian community, this photo essay examines our initial misinterpretation of a *bandal* project as successful locally‐led adaptation, and our subsequent reinterpretation of it as a configuration of three interrelated forms of responsibility: ‘self‐responsibility’, wherein at‐risk communities act under constraint; ‘passive responsibility’, manifested through fragmented expert and institutional knowledge; and ‘reactive responsibility’, embedded in public resource distribution patterns reflecting a logic of impact‐triggered humanitarian aid that constrains adaptive potential. We argue that, in the absence of active and proactive responsibilities assumed by a range of local actors, self‐responsibility is coerced, responsibilising at‐risk people and producing maladaptation. Locally‐led adaptation, therefore, ought to move beyond a solely community‐based framing towards a collectively accountable process.

## 1 INTRODUCTION

The term ‘locally‐led adaptation’ (LLA) consists of two promising concepts: local and adaptation. ‘Adaptation’, as a set of proactive decisions and actions by individuals and societies, constructs a way of tackling anticipated and experienced risks. The qualifying term ‘local’ highlights the agency of actors and institutions within affected societies in addressing those risks. As Oxley (2013, p. 3) noted: ‘vulnerable people when exposed to multiple shocks and stresses…adopt self‐help and mutual assistance strategies that are holistic, flexible, iterative and responsive to change’. Over recent decades, LLA has been promoted and mainstreamed into non‐governmental organisation (NGO) projects broadly categorised under climate change action. Some interpret LLA as an advancement from community‐based adaptation, explicitly reasserting the position of at‐risk people as best situated to develop adaptation strategies reflexive of specific needs and contexts (Westoby et al., 2020, 2021). The widely acknowledged value of LLA arises from leveraging indigenous knowledge, locally available resources, and grassroots leadership (Soanes et al., 2021; Rahman et al., 2023).

Its conceptual development is built on the fertile ground generated by earlier attention to a community‐based approach to adaptation, capitalising on a community's ‘priorities, needs, knowledge, and capacities’ above those of experts who exercise more power through governance institutions (Reid et al., 2009, p. 13). The LLA framework extends this ethos and seeks to incorporate a wider spectrum of local institutions beyond the narrow confines of grassroots‐level interventions, reclaiming the principles of community‐based strategies that have been co‐opted by practitioners (Vincent, 2023). The drive to understand and implement adaptation within complex political dynamics continues to inform contemporary efforts towards transformational adaptation.

Our first fieldwork trip, conducted in February 2023 among disaster‐affected communities, explored a case of a bamboo wall construction project initiated by an at‐risk community in Kurigram District, northern Bangladesh. Houses, schools, and the market in the village faced direct exposure to the eroding bank along the Brahmaputra River, leading to displacement. Coupled with severe poverty, the village is often cited as ‘most vulnerable’ to the devastating impacts of frequent floods, erosion, and displacement. The community‐initiated bamboo wall project embodied the principles of LLA, specifically through the utilisation of local knowledge, resources, and leadership as a means of self‐protection against riverbank erosion. However, upon revisiting the village in October 2023, immediately after the monsoon season, we observed the complete disappearance of the bamboo structure and significant erosion of residential land, resulting in displacement. This observation prompted a critical reflection on our previous assumptions regarding the construction as an LLA practice. Through an in‐depth evaluation of lived experiences and perceptions across diverse social and geographical positions within the community, we reinterpreted the case as locally‐led maladaptation (LLMA)—sustaining or exacerbating the risk of disaster and displacement.

This ethnography contributes to the ongoing discussion by critically examining the phenomenon of LLMA—which receives limited scholarly attention—with reflections on the authors' own positionality and earlier conceptual misjudgements. Our account of misinterpretation particularly centres on the romanticisation of the ‘local’, wherein the capacity of communities is co‐opted in ways that ultimately serve status quo solutions. This framing frequently overlooks the fundamental idea of adaptation, namely, to address and redress pre‐existing inequalities and structural vulnerabilities within society (Rahman et al., 2023; Vincent, 2023; see also Titz, Cannon, and Krüger, 2018; Melis and Apthorpe, 2020). The shift from misinterpretation to reinterpretation, as this ethnography explores, emerges from critical reflection on how LLA is problematically imagined: it places disproportionate responsibility on communities, while the passive responsibility of other local actors such as public officials, NGO practitioners, and civil society entities remains insufficiently problematised. Thus, our argument is to reclaim ‘locally‐led’ as a form of reconfiguring the active and proactive responsibility of various actors by challenging the overemphasis on a community's self‐governance, which is formed through the inaction of non‐community members in society.

This article delves into maladaptation with a theoretical focus on its inadvertent consequence of increasing disaster risks (section 2). Our primary objective is not to undermine locally embedded values or LLA per se; rather, we aim to analyse critically the social processes which distort these values, ultimately heightening vulnerabilities amid acute climate threats. Through an ethnography of a bamboo wall construction project, this study presents lived experiences and stories from the viewpoint of an at‐risk community. Using an ethnographic photo essay as a methodology facilitated a collaborative disaster study and co‐authorship (section 3). The unpacked case indicates that self‐responsibility of at‐risk communities initiates LLA, whereas the absence of active responsibilities in the preparatory phase renders the initiative maladaptive (section 4). We further discuss the configuration of responsibilities as a critical condition for LLA and LLMA; when self‐responsibility is coerced and forced upon at‐risk communities in the absence of shared responsibility, it contributes to the creation of disaster risk.

## 2 THEORETICAL CONSIDERATIONS

The Intergovernmental Panel on Climate Change (Field et al., 2014, p. 5) defines climate change adaptation as the planning and implementation ‘process’ for the ‘adjustment to actual or expected climate and its effects’. Yet, adaptation is far from a neutral process; it is inherently political, shaped by negotiations and struggles among actors with competing values, interests, and worldviews (Pelling, 2011; Hügel and Davies, 2020; Paprocki, 2021; Nyberg, Wright, and Bowden, 2022). Eriksen, Nightingale, and Eakin (2015, p. 531) represent the politics of adaptation as ‘struggles over authority, knowledges and subjectivities across scales by multiple actors’. A notable implication of this politics on governing adaptation is the hampered participation of less powerful actors within society (Mees and Driessen, 2019; Communications Earth & Environment, 2023). In Bangladesh, this exclusion is intertwined with pre‐existing power asymmetries structuring unequal participation in and the benefits of adaptation processes (Ishtiaque et al., 2021).

Despite recognition of these power asymmetries, the praxis of adaptation has largely remained technocratic, prioritising engineered measures and temporal achievements in adaptive capacity development (Ojha et al., 2016; Kehler and Birchall, 2021; Mills‐Novoa, 2023). Such a technocratic stance systematically distracts adaptation from addressing the root causes of disaster risks—broadly referred to as vulnerability (Ayers and Dodman, 2010; Kirkby, Williams, and Huq, 2018). Barnett and O'Neill (2010, p. 211) superimpose this notion on to the concept of maladaptation, denoting it as ‘action taken ostensibly to avoid or reduce vulnerability to climate change that…increases the vulnerability of…social groups’. In the same vein, critical scholarship on adaptation accords attention to increased vulnerability as the primary indicator of maladaptation (see, for example, Magnan et al., 2016; Schipper, 2020; Bertana et al., 2022). Thus, maladaptation is not simply a failed outcome, but a product of political and institutional processes that maintain and exacerbate risks.

Disaster studies further develops this line of thinking by introducing the concept of risk creation. Dickinson and Burton (2022, p. 203) explain that disaster risk creation is ‘a process that increases vulnerability’, while reduction of the risk is linked to responsibility and accountability based on the belief that disaster is not made by a superficial power but by the choices of humans and society (see also Kelman, 2022). The concept of risk creation also reorients the concern from ‘how do we adapt to climate change?’ to ‘how do we maladapt, thereby creating disaster risk associated with climate change?’, as a way of challenging an overemphasis on actions but rarely questioning their outcomes or inactions. Politics, as a lens with which to interrogate the unequal power relations of actors and the unjust decision‐making processes which benefit social actors disproportionately (Benn, 1960; Lukes, 2004; Gaventa, 2020), offers an analytical framework with which to unveil the discursive power and structured inequality that hamper the attainment of adaptive goals and the ability to address disaster risk effectively.

Balancing the discourse of adaptation with that of maladaptation—and disaster risk reduction with disaster risk creation—is complex, shaped by dominant knowledge hierarchies that often privilege the interpretations of powerful actors. As the theory of positionality reminds us, knowledge is ‘always’ situated in and shaped by the specific social position of the knower and embedded within the existing power structure (Collyer et al., 2019; Marguin et al., 2021; Singh, 2021). Accepting this, a study of (mal)adaptation requires us to ask who is subordinated to whom through these dynamics, and whose experiences remain systematically obscured under dominant celebrational narratives of adaptation. The exploratory and reflexive power of ethnography offers a means to reorient understanding from the perspective of those most at risk, as discussed further in the next section.

## 3 METHODOLOGY: ETHNOGRAPHIC PHOTO ESSAY

This study originated in in‐situ research of humanitarian governance for climate‐related displacement in Kurigram District, northern Bangladesh. The *National Adaption Plan of Bangladesh (2023–2050)* (GoB, 2022a) categorises the area as being prone to flood and riverbank erosion, with the *Bangladesh Disaster‐Related Statistics BDRS 2021* report (GoB, 2022b) indicating that 97,316 households were affected between 2015 and 2020. These disaster impacts are mainly intertwined with two broader contexts. Geographically, the region is bounded by the Brahmaputra, Teesta, and Dharla Rivers, while numerous smaller rivers and seasonal water bodies render the land highly exposed to annual flooding and erosion. In terms of the socio‐economic context, 70.87 per cent of the population live below the poverty line, and 2,026,591 individuals relied on loans between 2015 and 2020 (GoB, 2022b). The monsoon season, which typically occurs in June and July, is particularly destructive, as illustrated in this photo essay.

This article focuses on our fieldwork research into the construction of a bamboo wall in Begumganj Union, Ulipur Upazila subdistrict. The Union consists of 21 villages located along the Brahmaputra River and is recognised as the poorest area in the subdistrict. The bamboo wall was intended to protect Mollarhat bazaar (the local term for market), which supports villages across Begumganj Union in sustaining their livelihoods. We conducted our fieldwork in two phases in 2023. It was carried out mainly through applied participant observation, interviews, and informal conversations with diverse local actors in public spaces such as markets, homes along the riverbank, and government offices, as well as among NGOs operating in locations where disaster and displacement risks are experienced and discussed. We carried out data collection and analysis in the studied area, thereby identifying where we could gather more information to deepen our understanding. We continued gathering data until we reached saturation point and were unable to generate further significant insights (Bryant, 2020; Charmaz and Thornberg, 2021). In addition to 31 recorded interviews and extensive field notes, approximately 300 photographs taken at the site were not simply visual descriptions or evidence of our research activities, but also served as critical tools for reflection and shaped our interpretation both during the fieldwork and in the later stages of analysis and writing.

This study is primarily grounded in a process of reflexivity, particularly concerning our initial misinterpretation and subsequent reinterpretation of the case study. During our preliminary analysis through ongoing dialogue between the two authors at the study site, we reflected critically on our assumptions and promoted a rethinking of how we initially framed the project as an instance of successful adaptation but later came to understand as one of maladaptation. Central to this reflexive process was a judicious examination of how researchers' socio‐cultural backgrounds, prior knowledge, initial interests, and values shape the research process and findings (Walsh, 2003; Kunz, 2023). Two guiding questions anchored this inquiry: why did we misinterpret the case?; and what can we learn from our misinterpretation?

In the post‐fieldwork phase, we systematically organised our preliminary analyses based on field notes and revisited recorded interviews. This analytical process was carried out in parallel with the writing of this article. However, the writing stage in our collaborative approach raised the question of whether a formalised academic writing style served both authors equally. While the first author was more familiar with traditional academic conventions through training, the second author's critical and analytical engagement with the case was constrained by these stylistic expectations. Building on the premise that writing—particularly in the sphere of ethnographic research—should be a continuous critical process that reflects lived experiences, cultural practices, and interactional dynamics observed in field sites, we actively sought an alternative writing style. This pursuit of co‐authorship was inspired by anthropologist Helena Wulff's (2021, p. 1) suggestion that alternative writing styles can ‘convey social life more accurately than conventional academic writing’, noting that ‘writing in such non‐academic genres can…inspire academic writing to become more accessible. Recent developments…include collaborative text production with interlocutors and artists’.

Our decision to present the research as an ethnographic photo essay reflects our shared affinity for photography as a medium to represent people, lived experiences, and phenomena observed in the field. In this study, the taking of photographs was a deliberate ethnographic act, visualising the stories shared by the research participants. Each photograph was preceded by a process of reflective interpretation in which we consciously selected subjects and angles that could convey situated narratives. Post capture, we routinely discussed our motivations and aims in taking specific photographs. Through these exchanges we collaboratively constructed meanings, enhancing mutual understanding of subtle symbols and values encountered in the field. Photography thus functioned both as a mode of inquiry and as a reflective device, enabling us to trace how our analytical focus shifted over time. In doing so, the visual archive also played a crucial role in the collaboration between the two authors in engaged ethnographic writing.

Reflecting on this method, we ensured at the writing stage that our co‐authorship became more equal and critical vis‐à‐vis interpreting and representing by taking three primary steps. First, we reviewed our repository of thousands of photographs taken during our fieldwork, ultimately selecting seven images through an elicitation session to reconstruct the lived experiences of participants confronting climate‐related displacement (see also Hermansen and Fernández, 2020). Second, in line with the ethnographic tradition, our writing emphasised an interpretation of everyday life through thick description. Third, we discussed stories by revisiting our field notes in an attempt to amplify the perspectives of at‐risk individuals, foregrounding their difficulties and stressors. Consequently, we explicitly positioned ourselves as storytellers, a role frequently embraced by ethnographers and anthropologists (Gottlieb, 2016). These methodological steps were integral to constructing ethnographic knowledge and illuminating locally embedded aspects of social and cultural life through collaborative co‐authorship.

This article uses the real names of locations, but participants' names are anonymised in accordance with ethnographic convention. The faces of the individuals photographed are blurred to protect their privacy.

## 4 A *BANDAL* INITIATIVE AND THE CONFIGURATION OF RESPONSIBILITY

### 4.1 Everyday life on the edge

মেঘের কোলে রোদ হেসেছে, বাদল গেছে টুটি । আহা, হাহা, হা ।

*The sun rays have started smiling on the lap of the clouds, and the rains have stopped. How beautiful is the scene!*

আজ আমাদের ছুটি ও ভাই, আজ আমাদের ছুটি । আহা, হাহা, হা

*Today is our holiday o friend, today is our holiday. How beautiful is the scene!*

**FIGURE 1:**
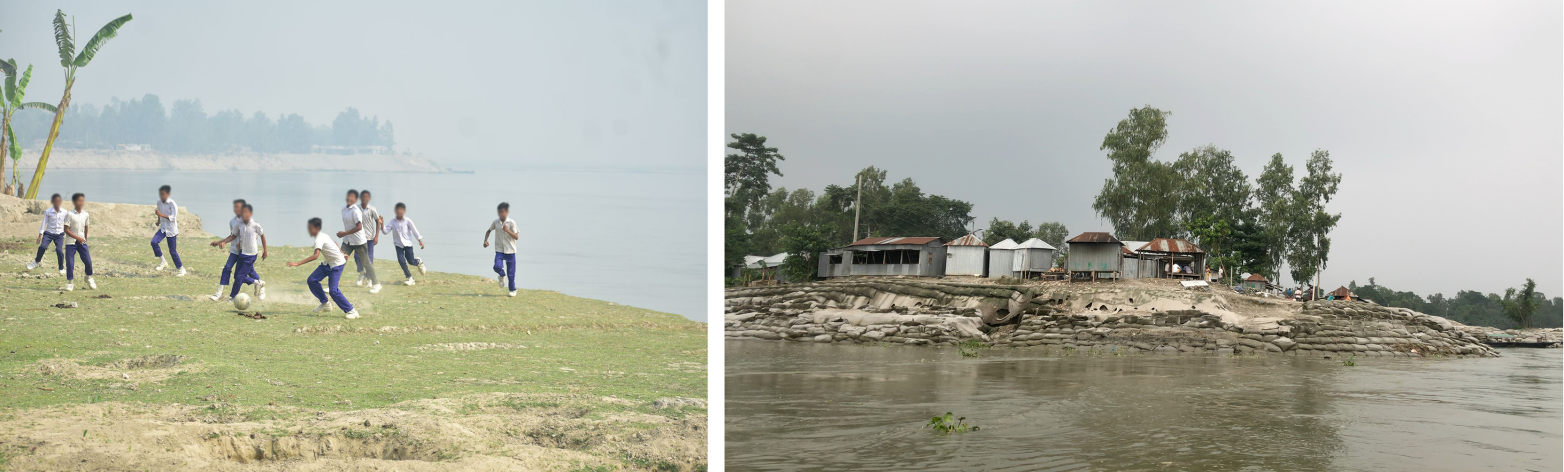
School and market on the edge of the eroding riverbank. **Source:** authors.

This evocative poem by Rabindranath Tagore vividly recalls the joy of childhood, depicting the simple pleasure of playing together in the beauty of the weather.^1^ We captured children's smiling faces in photographs, underlining that the playground is an indispensable space within their everyday school life with friends (see Figure 1). However, during our initial visit in February 2023, the villagers anticipated that the whole school compound would soon succumb to the encroaching Brahmaputra River in the imminent monsoon season. Their anticipation proved accurate. On a subsequent visit six months later, we could no longer locate the playground—indeed, even the school building had vanished. Children were displaced from the playground where they had once laughed and ran freely with friends—scenes poetically celebrated by Tagore. This stark reality prompted a cautious reflection by the authors on displacement, emphasising the profound loss of valued daily experiences. The Brahmaputra River had gradually consumed the fabric of everyday life, slowly eroding spaces until nothing remained.

The equivalent *playground* for adults is the Mollarhat bazaar. The local market serves as the economic lifeline for families. Fishers, farmers, and homemakers gather there, amidst the smell of fresh products and local food. More than just a site of economic life, it is also a place of social connection among villagers. The picture taken from the river shows how villagers' everyday livelihoods are on the verge of disappearance, and hence their personal linkages. But people are inherently adaptive. The layers of sandbags meticulously piled along the riverbank highlight villagers' persistent struggle to safeguard their socio‐economic spaces, juxtaposed poignantly with the visible limitations of adaptation, exemplified by the torn and ruptured defences.

As outsiders, when we first visited the Mollarhat bazaar, we were warmly welcomed with a hot cup of tea. The tea stall, colloquially known as ‘Cha Er Dokan’, functioned as the hub for knowledge exchange. Here, our interactions with villagers were reciprocal, enabling mutual learning and the sharing of experiences. During conversations at the tea stall, villagers shared information about numerous other villages, schools, and bazaars that had once existed between their village and the river, until they were lost to erosion. Drawing on their narratives, we reconstructed the meaning of adaptation in the context of displacement as a persistent struggle against the pace of gradual erosion of everyday life on the edge of the embankment.

### 4.2 Self‐responsibility

**FIGURE 2:**
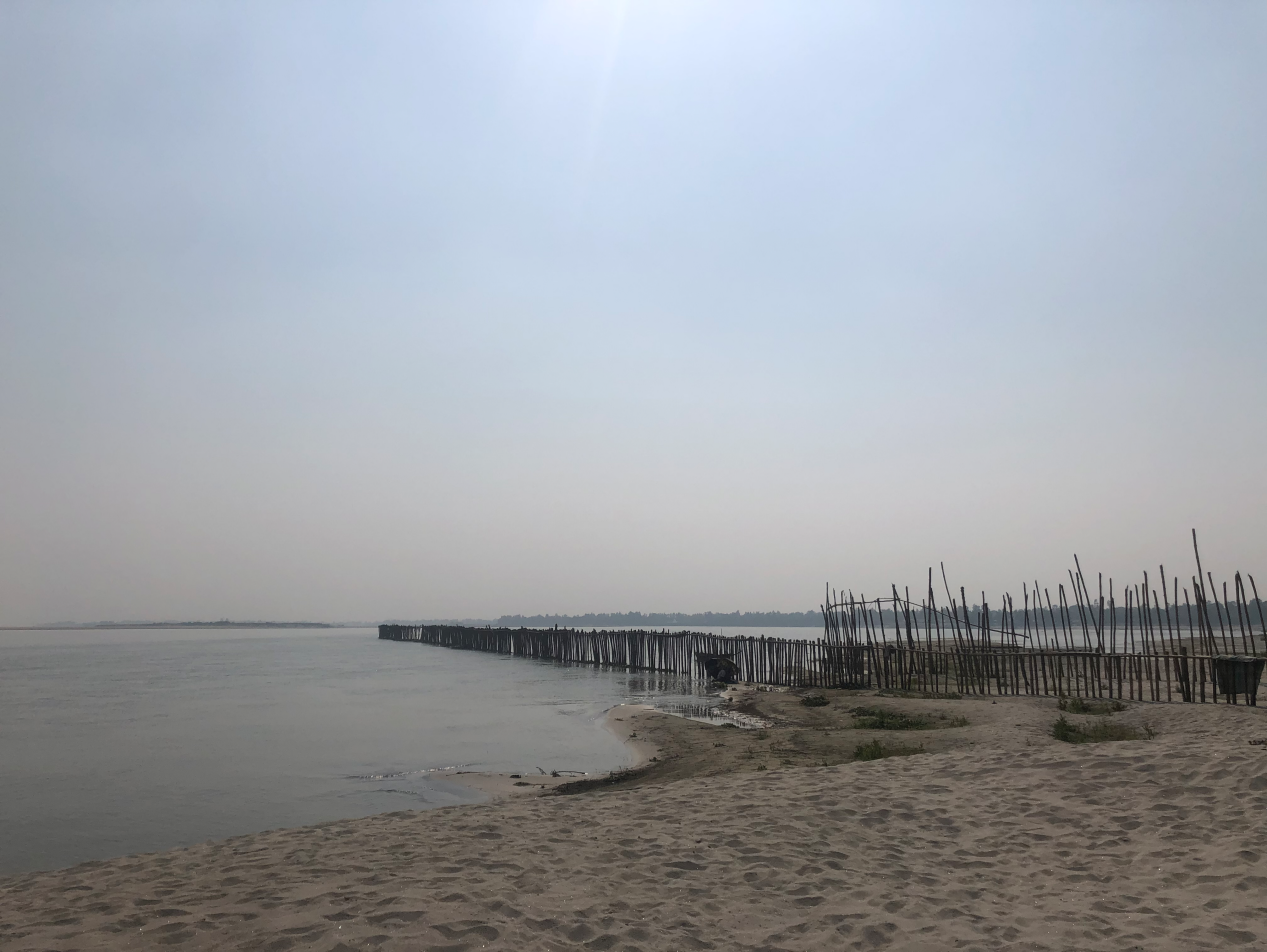
The *bandal* made by the local community on the Brahmaputra River. **Source:** authors.

In response to the erosion, the villagers decided to work together and take measures to protect those places in which their everyday life is rooted. Muhammad, an elected Member of Union Parishad, representing villagers at the lowest administrative level in the area under review, spearheaded the formation of an executive committee to draw up and implement the villagers' adaptive efforts. The committee decided to build a bamboo wall (see Figure 2), locally known as a *bandal*, a strategy learned from a neighbouring village where the construction of one had been part of an NGO project in the community to redirect and moderate a river current. The committee led by Muhammad first opted to secure funding of BDT 50,000 from Union Parishad.^2^ Recognising that this amount was insufficient, the committee selected representatives from each village in Begumganj Union to gather donations totalling BDT 200,000 from households. These representatives also organised fundraising during Hat days—twice‐weekly market gatherings where villagers sell and buy goods—eventually raising another BDT 50,000. The executive committee thus finally collected a total of BDT 300,000.

The *bandal* project required more than 1,800 bamboo sticks, each costing on average BDT 70. To position the bamboo, it was necessary to engage the services of a contractor, who charged BDT 80 per placement. Furthermore, the villagers were responsible for transporting the bamboo and carrying out other manual labour on a voluntary basis. As the project progressed, unforeseen expenses emerged, inflating the total project cost to BDT 432,000. Muhammad personally contributed the additional BDT 132,000, a sum neither the villagers nor the government could provide at short notice. Despite assurances from Upazila Parishad (the administrative unit of the subdistrict) that Muhammad's personal expenditure would be reimbursed, he has yet to receive any compensation. The committee also approached a prominent NGO in the district to request financial support to extend the bamboo wall before the monsoon season. The NGO agreed, citing previous successes with bamboo structures in other communities. It also expressed a willingness to continue financially supporting the expansion, which it does to date.

The community constructed the *bandal* in Falgun, the Bengali month falling between mid‐November and December, in 2022. At the site where the *bandal* stood—as a symbol of the autonomy of the community based on the collective commitments of villagers and leaders—we, as external researchers, initially interpreted the entire initiative as LLA. Central to this interpretation was the notion of *self‐responsibility*, with both community members and leaders actively engaged in addressing the risks they faced. This form of responsibility became more pronounced as the irresponsibility of other actors—evident in broken promises and institutional disengagement—pushed the community into a position of having to act alone.

### 4.3 Passive responsibility

In October, during a follow‐up field visit after the monsoon, our initial interpretation proved to be uncritically optimistic and overly romanticised. The bamboo structure had been washed away entirely, leaving no trace of where it had originally stood. While the community assumed that the *bandal* would lessen erosion‐induced displacement for several years—at least until the local government constructed the concrete embankment that they had long demanded—the outcome stood in stark contrast to this expectation: the structure collapsed in less than a year. The project, despite relying on local resources, knowledge, voluntary contributions, and leadership, ultimately failed in its adaptive goals. As a result, houses, a school, and other buildings vital in everyday life had either already suffered significantly owing to the consequences of erosion or were in danger of future erosion. We reinterpreted this outcome as LLMA, reflecting that substantial financial, intellectual, human, and temporal resources might have been better deployed through alternative adaptive strategies. We questioned our own enthusiasm in prematurely labelling the *bandal* story as LLA based solely on the visible bamboo wall structure and anecdotal narratives, while neglecting to carry out a deeper analysis of the nuanced realities of adaptation and maladaptation as a process of creating and reducing disaster risk.

**FIGURE 3:**
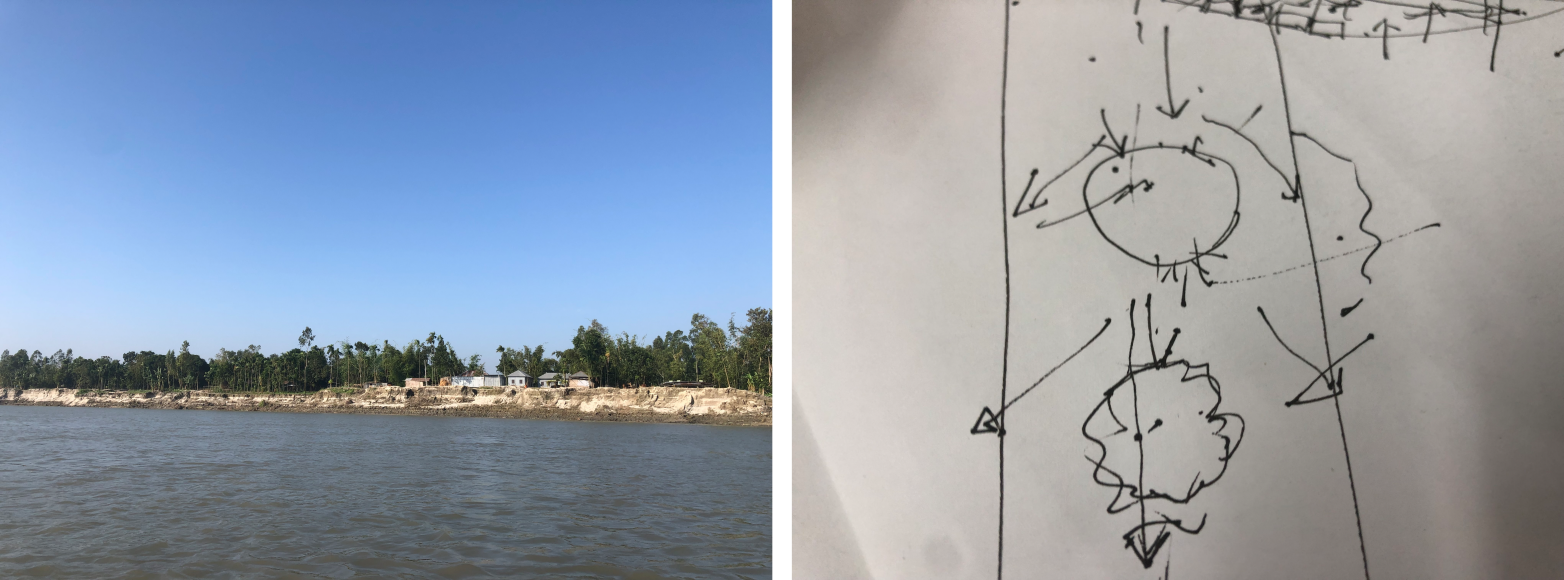
Further erosion and the engineer's sketch. **Source:** authors.

A conversation with an engineer from the government agency overseeing water resource management in the region elucidated the physical principles underlying the maladaptive consequences of the *bandal*. Illustrating his point with a sketch, he explained that rather than mitigating river currents, the bamboo structure could inadvertently redirect flows, creating whirlpools and intensifying the water's momentum, thereby exacerbating the erosion of the riverbank (see Figure 3). From an engineering standpoint, the *bandal* clearly exemplified maladaptation. Indeed, technical studies and reports support the engineer's view that a bamboo wall is effective in lessening the risk of floods or erosion in small water streams, but not in larger water bodies such as the Brahmaputra River (Ali, 2024).

The village's executive committee thus misapplied the experience of the NGO‐supported bamboo wall project in a neighbouring village to an unsuitable riparian context in their own locality. This misapplication might have been avoided had there been functional communication between the committee and the government agency's engineer. In this regard, the latter remarked that the villagers neither sought nor appeared receptive to his technical advice. He further noted that, even if he had been informed about the *bandal* initiative in time to offer his opinion on its potential to increase risk, he doubted whether the villagers would have listened to him. Conversely, the committee did not consider consulting the engineer, whose technical expertise—ideally exercised in a responsible manner—should have been mobilised to protect the community from water‐related hazards and disasters. This shows that knowledge around LLA and LLMA is deeply entangled with trust between villagers and external actors, shaping both the opportunities for and the barriers to communication that determine whether the intellectual capacities of diverse local experts are utilised responsibly within the locale.

In our reinterpretation of the *bandal* project as LLMA, the demolished wall came to symbolise the convergence of *self‐responsibility*—seen in the committee's attempt to replicate a model observed in a neighbouring village—and *passive responsibility*—embodied by local bureaucrats whose engineering expertise, while highly relevant, remained unshared. Operating within the constraints of institutional mandates, bureaucrats are not required to offer advice unless formally consulted. Yet this very system—one that enables actors to remain passive even when their knowledge could prevent failure—emerges as a structural source of the maladaptation we observed.

### 4.4 Reactive responsibility

**FIGURE 4:**
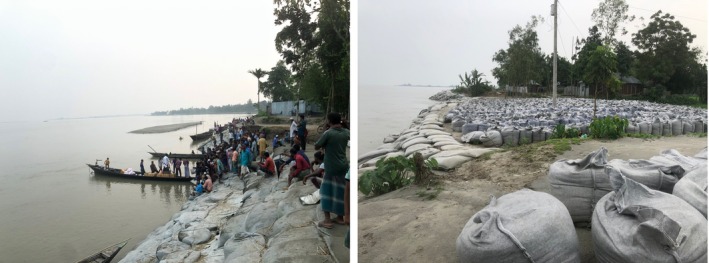
Food and sandbags provided by the government. **Source:** authors.

One evening, after a long day of field visits, we found ourselves walking along the riverbank back towards the bazaar. The river was in the glow of the setting sun. A crowd had gathered at the reddish edge of the river, some standing, others crouching, their eyes fixed on a boat moving steadily towards the shore. It was carrying sacks of rice, lentils, sugar, and cooking oil. The air was heavy with anticipation, for the boat carried the remedy for the weight of uncertainty. One villager explained that the boat transporting the goods was a component of a programme launched by the Trading Corporation of Bangladesh (TCB) to support low‐income families. As part of its responsibility to safeguard the everyday lives of adults and children, this government programme provides a TCB Family Card to people struggling to survive and earn a living, allowing them to purchase essential items at subsidised prices (see Figure 4).

Beyond the TCB, public assistance for displaced people includes emergency aid, building protective walls, placing sandbags along the riverbank, or relocation schemes such as the Ashrayan Project for displaced and landless populations. Villagers with whom we spoke acknowledged these forms of support but pointedly emphasised issues regarding their uneven and insufficient application.

During our initial visit to the village, parts of the area, containing, for example, houses, trees, the school, and the bustling playground, were still intact. Yet villagers were already urgently seeking protection of the riverbank, urging the government to reinforce the embankment with sandbags (locally termed geo‐bags) if concrete structures were not permitted. Their demands were communicated through petitions, direct visits to the local government offices, and appeals to local politicians. Persistent government inaction led the community to organise several human chain advocacy events, physically linking arms in solidarity, with village leaders actively participating. Local media representatives were invited to amplify the villagers' call for embankment reinforcement to prevent further erosion and displacement. Villagers noted, however, that government officials and politicians only came to measure the area of erosion (much like researchers) and took no meaningful action. Despite demanding proactive action for protection from erosion, local government authorities maintained a silence. One participant succinctly summarised the situation during a group discussion: ‘There's nothing else we can do. The UP [Union Parishad] Chairman and Members [referring to elected village representatives] are aware of our plight, but our requests for embankment construction go unanswered. Eventually, our only option is to become economically prepared for future erosion and displacement’.

During our second visit, six months after the monsoon season, the landscape had changed dramatically. As noted, the area where the school and homes had once stood had been swept away, leaving only rows of sandbags waiting to be laid out. But by then it was too late: the damage had already been done and people had been uprooted. Paradoxically, government support aimed at erosion prevention arrived only after the monsoon had resulted in displacement. This is partly due to the government's aid norms, which operationalise assistance based solely on observable and measurable—hence reportable—damage, thereby reinforcing a lingering *reactive responsibility*. Indeed, our empirical insights from the field suggest that such delays are caused primarily by a bureaucratic system that prioritises the allocation of public resources based on reported impacts rather than anticipated or ongoing risks.

This observation prompted a simple question: what if available resources had been allocated earlier, before community areas were eroded?

## 5 DISCUSSION

This ethnographic photo essay, grounded in the perspectives of diverse local actors, represents LLA as a socially constituted configuration of responsibility. The principal lesson arises from revisiting the authors' initial misinterpretation of the *bandal* as a good example of LLA, which stemmed from an overly narrow focus on the self‐responsibility of an at‐risk community. The proposed reinterpretation of LLA broadens the understanding of ‘local’ in adaptation projects as an assemblage of responsibilities collectively exercised by interdependent actors—including local government, NGOs, and other civil society entities—rather than by the community alone. LLA is widely framed as a self‐evident outcome of community action and its inherent potency (Rahman et al., 2021; Nath, 2024). This article, however, sheds light on the overlooked dimension of the shared responsibility of diverse local actors in mobilising knowledge and financial resources to ensure that LLA achieves its goal of protecting people from disaster and displacement.

The visual and narrative accounts from this ethnography show how self‐responsibility is coerced to offload adaptation burdens on to those least resourced to bear them. Importantly, this analysis does not interpret maladaptation as the result of individual irresponsibility given that the politics of responsibilisation hampers efforts to construct forward‐looking collective responsibility (Young, 2011; Trnka and Trundle, 2014). This notion is further illuminated by Khan's (2023) observation that neoliberal decentralisation imposes responsibilities on local governments in Bangladesh without adequate institutional development, thereby merely shifting the burden. Taking these insights into account, we discuss two possible ways of generating shared responsibility for LLA, in contrast to the prevailing tendencies of responsibility abdication.

On the one hand, understanding LLA as a relational process of creating responsibility is pivotal. When adaptation is approached as only being based on formal mandates and institutional duties, bureaucrats and local experts may default to passive responsibility, shaped by rigid institutional logics that inhibit discretionary and responsive practice to meet the needs of communities in the aftermath of displacement. Relation‐oriented reconfiguration of responsibility resonates with building accountable relationships and networks through which responsibility can be sought and co‐constructed rather than imposed through formal mechanisms (Moncrieffe, 2011; Aijazi, 2022; Ji, 2025). LLA is then no longer reduced to a project or community effort but becomes a process‐driven endeavour that hinges on the relational exercise of active engagements by diverse local actors.

On the other hand, shifting from an institutionalised norm of reactive responsibility towards a proactive form requires not just regulatory reform but also a broader cultural transformation. An anticipatory ethos of adaptation emerges through gradual and incremental changes in individual and collective perceptions, attitudes, and beliefs (Eriksen, Nightingale, and Eakin, 2015; Nightingale, Gonda, and Eriksen, 2022). This study identified a gap in the adaptation culture between community and government. Recent anthropological studies in Bangladesh contend that this lacuna is mainly due to the dominance of external knowledge in adaptation discourse and practice, whereby formal institutional actors in government and NGOs systematically overlook the interests and perspectives of the communities that they are meant to serve (Paprocki, 2021; Dewan, 2023). From this perspective, LLA ought to engage critically with unlearning externally‐driven adaptation culture to pursue reciprocal processes of exchange, listening, learning, and mutual articulation of each other's knowledges on equal terms within the locality. We contend that this is the realistic entry point for LLA and for preventing LLMA.

Addressing the limitations of this study is crucial for future research. Theoretically, this article did not interrogate the inherent limits of adaptation in reducing disaster risk. The political theory of ‘Climate Leviathan’ illuminates this limitation by suggesting that dominant responses to climate change increasingly reflect an aspiration for planetary management under capitalist hegemony, rather than a transformative rethinking of power and justice (Wainwright and Mann, 2013). From this stance, adaptation only offers a temporary resolution of managing risks, while underlying vulnerabilities and inequalities persistently create disaster risks. Moreover, while this article introduces the notion of shared responsibility as a way forward, it is limited in addressing the critical question of who holds the power to ensure that responsibility is shared meaningfully among multiple local actors and institutions in equitable ways for LLA. Methodologically, despite efforts to advance an interactive disaster study by centring the perspectives and narratives of at‐risk people, this ethnographic inquiry did not pursue a decolonial approach. To challenge research and practice that rank external knowledge over affected people's lived understandings of disaster and displacement risk (RADIX: Radical Interpretations of Disasters, n.d.; Gaillard, 2021; Alburo‐Canete, Alejandria, and Kreyscher, 2025), future research designed and conducted by at‐risk communities themselves could further advance—or even critically challenge—insights drawn from this study.

## Data Availability

The data that supports the findings of this study are available in the supplementary material of this article.

## Endnotes

^1^
This is part of the poem and song *Megher Kole Rod Heshechhe* (also known as *Chuti*), published in Bhadra 1315 (Bengali calendar, corresponding to August–September 1908). For more information, see: https://www.geetabitan.com/lyrics/M/megher-kole-rode-hesechhe-lyric.html (last accessed on 27 January 2026).

^2^
BDT 100,000 is equivalent to approximately USD 820.

## References

[disa70044-bib-0001] Aijazi, O. (2022) ‘Why technocratic understandings of humanitarian accountability undermine local communities’. Development in Practice. 32(2). pp. 175–187.

[disa70044-bib-0002] Alburo‐Canete, K.Z. , M.C. Alejandria , and K. Kreyscher (2025) ‘Guest editorial: advancing reflexive, creative and critical research methodologies for disaster studies’. Disaster Prevention and Management. 34(1). pp. 1–8.

[disa70044-bib-0003] Ali, M.S. (2024) ‘Bamboo bandalling technique for river bank protection and flood control – a critical review’. Current World Environment. 19(1). pp. 22–34.

[disa70044-bib-0004] Ayers, J. and D. Dodman (2010) ‘Climate change adaptation and development I: the state of the debate’. Progress in Development Studies. 10(2). pp. 161–168.

[disa70044-bib-0005] Barnett, J. and S. O'Neill (2010) ‘Maladaptation’. Global Environmental Change. 20(2). pp. 211–213.

[disa70044-bib-0006] Benn, S.I. (1960) ‘“Interests” in politics’. Proceedings of the Aristotelian Society. 60(1). pp. 123–140.

[disa70044-bib-0007] Bertana, A. , B. Clark , T.M. Benney , and C. Quackenbush (2022) ‘Beyond maladaptation: structural barriers to successful adaptation’. Environmental Sociology. 8(4). pp. 448–458.

[disa70044-bib-0008] Bryant, A. (2020) ‘The grounded theory method’. In P. Leavy (ed.) The Oxford Handbook of Qualitative Research. Oxford University Press, Oxford. pp. 166–199.

[disa70044-bib-0009] Charmaz, K. and R. Thornberg (2021) ‘The pursuit of quality in grounded theory’. Qualitative Research in Psychology. 18(3). pp. 305–327.

[disa70044-bib-0010] Collyer, F. , R. Connell , J. Maia , and R. Morrell (2019) Knowledge and Global Power: Making New Sciences in the South. Monash University Publishing, Clayton, VIC.

[disa70044-bib-0011] Communications Earth & Environment (2023) ‘Effective climate adaptation must be imaginative and inclusive’. Communications Earth & Environment. 4(1). Article number: 481. 10.1038/s43247-023-01150-4.

[disa70044-bib-0012] Dewan, C. (2023) Misreading the Bengal Delta: Climate Change, Development and Livelihoods in Coastal Bangladesh. The University Press Limited, Dhaka.

[disa70044-bib-0013] Dickinson, T. and I. Burton (2022) ‘Disaster risk creation: the new vulnerability’. In G. Bankoff and D. Hilhorst (eds.) Why Vulnerability Still Matters: The Politics of Disaster Risk Creation. Routledge, Abingdon. pp. 192–205.

[disa70044-bib-0014] Eriksen, S.H. , A.J. Nightingale , and H. Eakin (2015) ‘Reframing adaptation: the political nature of climate change adaptation’. Global Environmental Change. 35 (November). pp. 523–533.

[disa70044-bib-0015] Field, C.B. et al. (2014) Climate Change 2014: Impacts, Adaptation, and Vulnerability. Summary for Policymakers. Cambridge University Press, Cambridge.

[disa70044-bib-0016] Gaillard, J‐C. (2021) The Invention of Disaster: Power and Knowledge in Discourses on Hazard and Vulnerability. Routledge, Abingdon.

[disa70044-bib-0017] Gaventa, J. (2020) ‘Applying power analysis: using the “powercube” to explore forms, levels and spaces’. In R. McGee and J. Pettit (eds.) Power, Empowerment and Social Change. Routledge, Abingdon. pp. 117–138.

[disa70044-bib-0018] GoB (Government of Bangladesh) (2022a) National Adaption Plan of Bangladesh (2023–2050) . October. https://moef.portal.gov.bd/sites/default/files/files/moef.portal.gov.bd/npfblock/903c6d55_3fa3_4d24_a4e1_0611eaa3cb69/National%20Adaptation%20Plan%20of%20Bangladesh%20%282023‐2050%29%20%281%29.pdf (last accessed on 14 January 2026).

[disa70044-bib-0019] GoB (2022b) Bangladesh Disaster‐Related Statistics BDRS 2021: Climate Change and Natural Disaster Perspectives. December. http://nsds.bbs.gov.bd/storage/files/1/Bangladesh%20Disaster-related%20Statistics%20BDRS%202021.pdf (last accessed on 14 January 2026).

[disa70044-bib-0020] Gottlieb, A. (2016) ‘The anthropologist as storyteller’. In H. Wulff (ed.) The Anthropologist as Writer: Genres and Contexts in the Twenty‐First Century. Berghahn Books, New York City, NY. pp. 93–117.

[disa70044-bib-0021] Hermansen, P. and R. Fernández (2020) ‘Photo‐ethnography and political engagement: a study of the performative subversions of public space’. Dearq. 26 (January). pp. 100–109.

[disa70044-bib-0022] Hügel, S. and A.R. Davies (2020) ‘Public participation, engagement, and climate change adaptation: a review of the research literature’. WIREs Climate Change. 11(4). Article number: e645. 10.1002/wcc.645. PMC9285715 35859618

[disa70044-bib-0023] Ishtiaque, A. et al. (2021) ‘Multilevel governance in climate change adaptation in Bangladesh: structure, processes, and power dynamics’. Regional Environmental Change. 21(3). Article number: 75. 10.1007/s10113-021-01802-1.

[disa70044-bib-0024] Ji, H. (2025) ‘Inter‐community participatory social network analysis: re‐envisioning humanitarian accountability with climate‐related displaced communities in Bangladesh’. Disaster Prevention and Management. 34(1). pp. 133–146.

[disa70044-bib-0025] Kehler, S. and S.J. Birchall (2021) ‘Social vulnerability and climate change adaptation: the critical importance of moving beyond technocratic policy approaches’. Environmental Science & Policy. 124 (October). pp. 471–477.

[disa70044-bib-0026] Kelman, I. (2022) Disaster by Choice: How Our Actions Turn Natural Hazards into Catastrophes. Oxford University Press, Oxford.

[disa70044-bib-0027] Khan, N. (2023) River Life and the Upspring of Nature. Duke University Press, Durham, NC.

[disa70044-bib-0028] Kirkby, P. , C. Williams , and S. Huq (2018) ‘Community‐based adaptation (CBA): adding conceptual clarity to the approach, and establishing its principles and challenges’. Climate and Development. 10(7). pp. 577–589.

[disa70044-bib-0029] Kunz, R. (2023) ‘Reflexive inquiry’. In M.B. Salter , C.E. Mutlu , and P.M. Frowd (eds.) Research Methods in Critical Security Studies: An Introduction. Routledge, Abingdon. pp. 74–78.

[disa70044-bib-0030] Lukes, S. (2004) Power: A Radical View. Palgrave Macmillan, London.

[disa70044-bib-0031] Magnan, A.K. et al. (2016) ‘Addressing the risk of maladaptation to climate change’. WIREs Climate Change. 7(5). pp. 646–665.

[disa70044-bib-0032] Marguin, S. et al. (2021) ‘Positionality reloaded: debating the dimensions of reflexivity in the relationship between science and society: an editorial’. Historical Social Research. 46(2). pp. 7–34.

[disa70044-bib-0033] Mees, H. and P. Driessen (2019) ‘A framework for assessing the accountability of local governance arrangements for adaptation to climate change’. Journal of Environmental Planning and Management. 62(4). pp. 671–691.

[disa70044-bib-0034] Melis, S. and R. Apthorpe (2020) ‘The politics of the multi‐local in disaster governance’. Politics and Governance. 8(4). pp. 366–374.

[disa70044-bib-0035] Mills‐Novoa, M. (2023) ‘What happens after climate change adaptation projects end: a community‐based approach to ex‐post assessment of adaptation projects’. Global Environmental Change. 80 (May). Article number: 102655. 10.1016/j.gloenvcha.2023.102655.

[disa70044-bib-0036] Moncrieffe, J. (2011) Relational Accountability: Complexities of Structural Injustice. Zed Books, London.

[disa70044-bib-0037] Nath, S. (2024) ‘Mobilising transformative community‐based climate change adaptation’. Urban Transformations. 6(1). Article number: 1. 10.1186/s42854-023-00059-7.

[disa70044-bib-0038] Nightingale, A.J. , N. Gonda , and S.H. Eriksen (2022) ‘Affective adaptation = effective transformation? Shifting the politics of climate change adaptation and transformation from the status quo’. *WIREs* Climate Change. 13(1). Article number: e740. 10.1002/wcc.740.

[disa70044-bib-0039] Nyberg, D. , C. Wright , and V. Bowden (2022) Organising Responses to Climate Change: The Politics of Mitigation, Adaptation and Suffering. Cambridge University Press, Cambridge.

[disa70044-bib-0040] Ojha, H.R. et al. (2016) ‘Policy without politics: technocratic control of climate change adaptation policy making in Nepal’. Climate Policy. 16(4). pp. 415–433.

[disa70044-bib-0041] Oxley, M.C. (2013) ‘A “people‐centred principles‐based” post‐Hyogo framework to strengthen the resilience of nations and communities’. International Journal of Disaster Risk Reduction. 4 (June). pp. 1–9.

[disa70044-bib-0042] Paprocki, K. (2021) Threatening Dystopias: The Global Politics of Climate Change Adaptation in Bangladesh. Cornell University Press, Ithaca, NY.

[disa70044-bib-0043] Pelling, M. (2011) Adaptation to Climate Change: From Resilience to Transformation. Routledge, Abingdon.

[disa70044-bib-0044] RADIX: Radical Interpretations of Disasters (n.d.) ‘Power, prestige & forgotten values: a disaster studies manifesto’. Website. https://www.radixonline.org/manifesto-accord (last accessed on 14 January 2026).

[disa70044-bib-0045] Rahman, M.F. et al. (2023) ‘Locally led adaptation: promise, pitfalls, and possibilities’. Ambio. 52(10). pp. 1543–1557. 37286919 10.1007/s13280-023-01884-7 PMC10460758

[disa70044-bib-0046] Rahman, T.H.M. , A. Albizua , B. Soubry , and W. Tourangeau (2021) ‘A framework for using autonomous adaptation as a leverage point in sustainable climate adaptation’. *Climate* Risk Management. 34. Article number: 100376. 10.1016/j.crm.2021.100376.

[disa70044-bib-0047] Reid, H. , M. Alam , R. Berger , T. Cannon , and A. Milligan (2009) Participatory Learning and Action 60: Community‐Based Adaptation to Climate Change. International Institute for Environment and Development, London.

[disa70044-bib-0048] Schipper, E.L.F. (2020) ‘Maladaptation: when adaptation to climate change goes very wrong’. One Earth. 3(4). pp. 409–414.

[disa70044-bib-0049] Singh, J.N. (2021) ‘Commentary: epistemological positionalities’. Applied Linguistics. 42(6). pp. 1168–1175.

[disa70044-bib-0050] Soanes, M. et al. (2021) Principles for Locally Led Adaptation: A Call to Action. Issue Paper. January. International Institute for Environment and Development, London.

[disa70044-bib-0051] Titz, A. , T. Cannon , and F. Krüger (2018) ‘Uncovering “community”: challenging an elusive concept in development and disaster related work’. Societies. 8(3). Article number: 71. 10.3390/soc8030071.

[disa70044-bib-0052] Trnka, S. and C. Trundle (2014) ‘Competing responsibilities: moving beyond neoliberal responsibilisation’. Anthropological Forum. 24(2). pp. 136–153.

[disa70044-bib-0053] Vincent, K. (2023) ‘Development geography II: community‐based adaptation and locally‐led adaptation’. Progress in Human Geography. 47(4). pp. 604–612.

[disa70044-bib-0054] Wainwright, J. and G. Mann (2013) ‘Climate Leviathan’. Antipode. 45(1). pp. 1–22.

[disa70044-bib-0055] Walsh, R. (2003) ‘The methods of reflexivity’. The Humanistic Psychologist. 31(4), pp. 51–66.

[disa70044-bib-0056] Westoby, R. , K.E. McNamara , R. Kumar , and P.D. Nunn (2020) ‘From community‐based to locally led adaptation: evidence from Vanuatu’. Ambio. 49(9). pp. 1466–1473. 31776968 10.1007/s13280-019-01294-8 PMC7320106

[disa70044-bib-0057] Westoby, R. et al. (2021) ‘Locally led adaptation: drivers for appropriate grassroots initiatives’. Local Environment. 26(2). pp. 313–319.

[disa70044-bib-0058] Wulff, H. (2021) ‘Writing anthropology’. In F. Stein (ed.) The Open Encyclopedia of Anthropology. 26 February. https://www.anthroencyclopedia.com/entry/writing-anthropology (last accessed on 14 January 2026).

[disa70044-bib-0059] Young, I.M. (2011) Responsibility for Justice. Oxford University Press, Oxford.

